# Bibliographical Notices

**Published:** 1842-03-05

**Authors:** 


// MEDICAL EXAMINER.

NEW SERIES.

No. 10.] PHILADELPHIA, MARCH 5, 1842. [Vol. I.

### BIBLIOGRAPHICAL NOTICES.

*The Practice of Medicine, or a Treatise on Special Pathology and Therapeutics.* By Robley Dunglison, M. D., Professor of the Institutes of Medicine, &c. in Jefferson Medical College, Philadelphia; Lecturer on Clinical Medicine, and Attending Physician at the Philadelphia Hospital, &c. &c. In two volumes, 8vo. Philadelphia, 1842.

This work supplies a want which has been long felt—an elementary treatise for students, which includes the recent additions which have been made to medicine. The older works, not excepting those published some ten or fifteen years since are all deficient in this respect, and we have no doubt that this new one will be received in a manner which will be highly gratifying to the author.

His views as to the manner in which the book is written are explained in the preface.

"In regard to the execution of the work, the author would merely remark, that he has endeavoured to give a faithful exposition of what he considered to be the existing views in relation to the subjects of which he treats. He is not conscious of possessing any exclusive opinions; and has endeavoured to be essentially eclectic. Neither is he aware of having any undue prejudices. It has been his good fortune to pass, thus far, through life without imbibing unpleasant feelings towards any honourable member of the profession; he has, accordingly, throughout the work felt a pleasure in referring to the labours of observers everywhere, and it has been no little satisfaction to him that he has been called upon so often to make mention of the investigations of those on this side of the Atlantic."

There are many advantages in the eclectic system of practice; to a greater or less extent most physicians of the present day are attached to no peculiar opinion or set of opinions. It does not necessarily bind the physician to the opinions adopted by him as the best for the present; they may change as new discoveries enlarge our circle of facts. The author is strictly eclectic; so much so, that he speaks always in the third person, and quotes opinions expressed by him in other publications, instead of simply repeating the expressions in the first person. We confess, as a matter of taste, we do not admire this peculiar mode; the ordinary form appears to us more simple, and therefore preferable.

The references in the work, as well as the formulæ, which are numerous, are included in the body of the text, and the author, with feelings of honourable justice to his professional brethren, has mentioned the different writers whose opinions he cites. This practice, although required in strict justice whenever quotations are admissible, is sometimes strangely forgotten by our own, as well as foreign medical writers.

The arrangement of the work is as follows. Each chapter is subdivided into its appropriate sections.

"Book I.—Diseases of the Alimentary Canal. Chapter 1.—Diseases of the Mouth. Chapter 2.—Diseases of the Pharynx and Œsophagus. Chapter 3.—Diseases of the Stomach. Chapter 4.—Diseases of the Intestines. Chapter 5.—Diseases of the Peritoneum. Chapter 6.—Morbid productions in the Peritoneum and Intestines.

Book II.—Diseases of the Respiratory Organs. Chapter 1.—Diseases of the Larynx and Trachea. Chapter 2.—Diseases of the Bronchia and Lungs. Chapter 3.—Diseases of the Pleura. Chapter 4.—Asphyxia.

Book III.—Diseases of the Circulatory Apparatus. Chapter 1.—Morbid conditions of the Blood. Chapter 2.—Diseases of the Circulatory Organs.

Book IV.—Diseases of the Glandiform Ganglions. Chapter 1.—Diseases of the Spleen. Chapter 2.—Diseases of the Thyroid Gland. Chapter 3.—Diseases of the Thymus Gland, and Supra-Renal Capsules. Chapter 4.—Diseases of the Mesenteric Glands."

A work of this kind requires more time for thorough preparation than can well be given to a first edition; we have no doubt that the second one will remove all traces of haste. We would apologise on this ground for the erroneous statement, that, "in the French hospitals, three times as many cases of pneumonia have been observed in the last six months of the year as in the first." The actual relation is widely different; of 296 cases in the French hospitals, the date of which was ascertained by Grisolle, only seventy-nine occurred in the six months beginning with the first of July. In our own country the disproportion of cases is also very great, many more occurring in the first than in the last six months of the year.

To the same cause we would attribute some errors in the estimate of the author as to the mortality from delirium tremens in the Men's Lunatic Asylum. He has copied the estimate of cases and results from the statements prepared by a nurse in the hospital, and handed by him to Dr. Pennock. The number of fatal cases in the year 1839 is stated to be six; it was really only three, and of these cases, one died from injuries received in leaping from a third story window; another was treated by opium before his entrance, and died soon after admission. The error, however, was natural enough for one not acquainted with the source of the documents. We regret it because an attempt to render therapeutics a more positive branch of medicine is always difficult, and should not be weakened, unless on very certain grounds.

Our limits do not permit us to enter upon an analysis of the work; we are obliged to limit notices of this character to monographs embracing a less wide range of subjeets. To the practitioner it will prove interesting, while it will furnish to the student—for whom it seems to be principally designed—a vast amount of useful, and, to the larger number, novel information. We are obliged to content ourselves with expressing the gratification the work has afforded us, and the belief that it will prove generally useful and acceptable.

---

*Quarterly Summary of the Transactions of the College of Physicians of Philadelphia*,—November and December 1841, and January 1842: pamphlet, pp. 22.

We cannot more appropriately introduce this little journal than by extracting a portion of the announcement with which it commences.

"The College of Physicians of Philadelphia was instituted towards the close of the year 1786; the first stated meeting, after its full organization, was held on the 2d of January, 1787. About two years afterwards an act of incorporation was obtained from the Legislature of Pennsylvania. It is believed to be the oldest College of Physicians in the United States, and among its founders and early members, it includes some of the most eminent physicians that our country has produced.

The objects of the association, as expressed first in the Constitution and afterwards in the preamble to the charter, are "to advance the science of medicine, and thereby lessen human misery, by investigating the diseases and remedies which are peculiar to this country; by observing the effects of different seasons, climates and situations upon the human body; by recording the changes which are produced in diseases by the progress of agriculture, arts, population, and manners; by searching for medicine in the American woods, waters, and in the bowels of the earth; by enlarging the avenues to knowledge from the discoveries and publications of foreign countries: and by cultivating order and uniformity in the practice of physic." In order more fully to accomplish these important objects—to encourage and stimulate its members to their more zealous pursuit, the College, agreeably to the recommendation of a committee, consisting of Drs. Condie, B. H. Coates, Parrish, Bond, Moore and Pepper, appointed at the stated meeting in June, 1841, resolved at the stated meeting in September ensuing, to publish a bulletin of their transactions, after the example of other scientific bodies, who have adopted the practice with so much advantage to their own interests, and that of science generally.—At the meeting in November, Drs. Condie, Parrish and Bell were appointed a committee to carry this resolution into effect. It is hoped that the result of adopting this course, will be to revive that spirit, which actuated the college in its earliest years—to encourage the number and value of the communications of its fellows—to prompt a more punctual attendance, and to insure to the institution, by more fully deserving it, that prominent place in the public respect and confidence to which it has heretofore been deemed to be entitled. There is no law in this State establishing a medical police, or regulating the practice of medicine; but so far as these functions have been exercised, it has been chiefly done by this College. It is the body to which reference has generally been made by the Governor of the Commonwealth, and other civil authorities, including the Board of Health, on the occurrence of alarming epidemics, and upon important questions of medical jurisprudence.

Considering the relation which Philadelphia holds to the rest of the country in regard to medical education, and the position in which this College is placed in relation to the medical profession of this; considering what similar institutions are doing in some parts of Europe, both honor and duty would seem to demand that this body should mingle more earnestly in the honorable competition, to add to the usefulness and lustre of the profession.

The College has been in operation fifty-five years."

Then follows a list of the officers from the first President, Dr. John Redman, to the present time.

The meetings of the College are monthly, and the report of the first session, Nov. 2d, 1841, consists chiefly of the annual report of the committee of Surgery on the progress of that science, by its chairman, Isaac Parrish, M. D.

This paper offers but few points for comment. It presents a rapid analysis of the views of M. Ricord, on the form and treatment of syphilitic affections, and comments upon the several operations of tenotomy and myotomy of Dieffenbach and others. We quote, with the most hearty concurrence a single passage of severe but perfectly just criticism.

"Within the past few years, the records of Surgery have been defaced by the most extravagant accounts of the success of tendon cutting, and other operations to cure deformities. Take the following heading as an example: "Subcutaneous section of forty two muscles, tendons or ligaments, practised the same day, on the same person, to cure a general articular deformity, by M. Jules Guerin," of Paris.

Cases have been reported as cures, long before sufficient time had elapsed, to pronounce them as such, according to the fixed laws of nature, and the legitimate deductions of reason. Thus it is, that the confidence of the profession in medical testimony is weakened, and the permanent progress of the science is impeded."

A short notice of the operation of Dr. John Rhea Barton, for anchylosis of the knee joint, and for recto-vaginal fistula, closes the article.

Dr. B. H. Coates read a paper on the moral grounds for the condemnation of Quackery, &c., and communicated verbally, a case of typhoid fever, with ulceration and discharge of fœcal matter from the umbilicus, without hernia.

The proceedings of the two succeeding meetings will be noticed in our next number.

---

*A Therapeutical Arrangement of the Materia Medica, etc.* By Martyn Payne, M. D., A. M., &c. New York, 1842. 18mo. pp. 271.

A well aranged syllabus of lectures, making a convenient and beautiful volume. It is intended, of course, for the students following the lectures on Materia Medica given by the author in the University of New York.

---

*Essay on Trembles, Milk Sickness, &c.* By John S. Seaton, M. D.

The disease is attributed by the author to arsenic dissolved in the springs from which the cattle drink. We observed that this explanation is not admitted by the editor of the Western Journal, who must be much more competent to decide on this matter than ourselves. It does not appear to us that the author has demonstrated his opinions in a satisfactory manner.

